# Self-Powered Z-Shaped Hybrid Triboelectric-Electromagnetic Vibration Sensor for Coal Mine Fracturing Condition Monitoring

**DOI:** 10.3390/mi17070786

**Published:** 2026-06-28

**Authors:** Yanping Miao, Da Liu, Zexu Zuo, Yanjun Feng, Chuan Wu

**Affiliations:** 1 Shaanxi Coal Caojiatan Mining Co., Ltd., Yulin 719100, China; yanpingmiao111@163.com; 2 Faculty of Mechanical and Electronic Information, China University of Geosciences (Wuhan), Wuhan 430074, China; 18872623924@163.com (D.L.); 18436099151@163.com (Z.Z.); 3 Tiandi Science and Technology Co., Ltd., Beijing 100013, China; 4 CCTEG Coal Mining Research Institute, Beijing 100013, China; 5 CCTEG (Xi’an) Mining Engineering Technology Co., Ltd., Xi’an 710000, China

**Keywords:** triboelectric nanogenerator, self-powered, vibration sensor, coal mine fracturing

## Abstract

During coal mine fracturing operations, real-time monitoring of the vibration frequency of the drilling assembly is crucial for assessing crack development, optimizing fracturing parameters, and ensuring the safety of downhole equipment. However, traditional active vibration sensors are limited by their reliance on external power supplies in the complex environment of underground mining, reducing their operational efficiency and effectiveness. Accordingly, a self-powered Z-shaped vibration sensor based on hybrid triboelectric and electromagnetic mechanisms was developed for monitoring coal mine fracturing drilling. This sensor utilizes the vibrations of the drilling tool to induce frictional electric pulse signals that correspond to the vibration frequency, enabling simultaneous vibration monitoring and energy generation. Experimental results demonstrate the stable performance of the proposed sensor under thermal conditions up to 150 °C and moisture levels reaching 90% relative humidity. The proposed sensor exhibits an operating frequency range of 0 to 11 Hz, with the measurement deviation constrained within a 5% threshold. Under optimal impedance matching, the triboelectric and electromagnetic units deliver peak power outputs of 0.04 mW and 110.5 mW when connected to external loads of 10^8^ Ω and 3.3 × 10^2^ Ω respectively. The proposed hybrid self-powered sensor uses the high-amplitude pulsed voltage signals generated by the TENG unit for vibration frequency identification, while the EMG unit harvests mechanical energy from low-frequency vibrations, thereby enhancing the self-powered capability of the sensor for underground vibration monitoring in coal-mine hydraulic fracturing drilling.

## 1. Introduction

Coal serves as a crucial pillar of the global energy system, playing an irreplaceable role in ensuring energy security and facilitating industrial production worldwide [1]. With the depletion of shallow resources, the global mining industry faces a collective challenge to transition to deeper strata, which presents extremely high geostress and complex gas outburst issues. In this context, hydraulic fracturing technology has emerged as a globally recognized key method for enhancing coalbed methane recovery [2], preventing rock bursts [3], and managing gas hazards [4]. Real-time monitoring of the vibrational characteristics of drilling tools during fracturing operations is of significant engineering importance for assessing fracture development [5], optimizing fracturing parameters [6], and ensuring the safety of downhole tools.

At present, underground vibration monitoring sensors mainly include electromagnetic geophones [7,8], piezoelectric accelerometers [9], capacitive MEMS accelerometers [10], and fiber-optic vibration sensors [11]. However, most of these sensors rely heavily on external batteries or cable-based power supplies, making it difficult to meet the requirements of modern mine safety monitoring for long-term, stable, and reliable operation. On one hand, chemical batteries are inadequate in terms of explosion-proof safety in high-gas environments in fracturing zones, and the high-humidity and -temperature conditions underground impose significant maintenance pressure due to the frequent battery replacements. On the other hand, wired monitoring systems incur high costs and lack flexibility when deployed over long distances and in complex geological conditions, and signals are easily disrupted by underground electromagnetic interference. Therefore, developing self-powered vibrational sensors that do not require external power sources is of great significance for achieving maintenance-free and highly reliable monitoring equipment.

To address the challenging issue of energy supply in complex environments, the concept of triboelectric nanogenerators (TENGs), first introduced by Wang, has found widespread application in self-powered sensing fields [12]. As an efficient energy conversion technology, TENGs can harness various forms of mechanical energy from both natural and industrial environments, such as vibrational energy [13], tidal energy [14], wave energy [15,16], wind energy [17,18,19], and hydropower [20,21,22]. In the realm of sensing and monitoring, self-powered sensors based on TENG have successfully captured a range of physical parameters, encompassing critical metrics such as humidity [23,24], temperature [25,26], frequency [27], displacement [28], tilt [29], rainfall [30], velocity [31,32], and pressure [33,34].

In recent years, thanks to its diverse structural designs, wide range of material selections, and sensitive low-frequency response, the triboelectric nanogenerator has gradually transitioned from fundamental research to specific engineering applications, demonstrating promising potential in environmental monitoring [35], landslide geological monitoring [36], and drill pipe rotational speed monitoring [37]. Evidently, TENGs are well-suited for the implementation of self-powered sensors. We herein present a Z-shaped vibration sensor for coal mine fracturing drilling, which integrates triboelectric and electromagnetic induction principles. By harvesting energy from ambient underground vibrations and simultaneously measuring vibration frequencies, the proposed device effectively addresses the long-standing power supply challenges for underground monitoring systems.

## 2. Structural Design and Operational Mechanism

**Figure 1:**
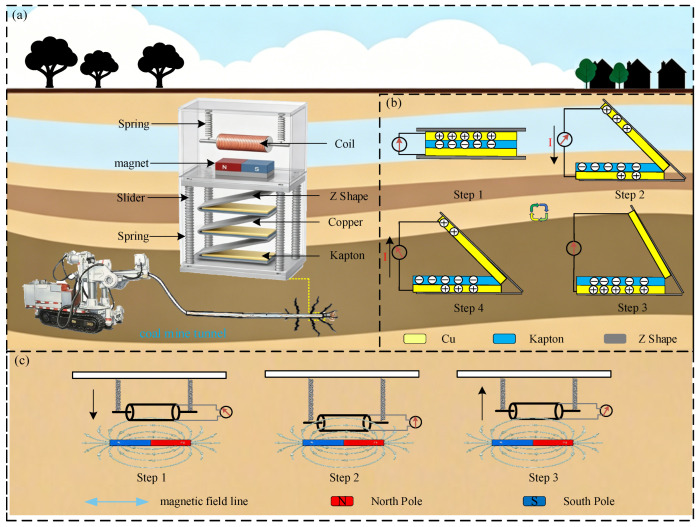
Schematic diagrams of the sensor. (**a**) 3D structural illustration; (**b**) Working principle of the TENG module; (**c**) Working principle of the EMG module.

The sensor features a sealed rectangular geometry, as illustrated in Figure 1a. Its external dimensions measure 128 mm × 97 mm × 197 mm. The proposed sensor integrates a triboelectric nanogenerator (TENG) combined with an electromagnetic generator (EMG). Specifically, the TENG unit is constructed with a housing, a trapezoidal base, a sliding mass, and springs. The sliding block, serving as an inertial mass, is sleeved on four vertically distributed guiding studs through reserved holes at its four corners. In coordination with the compression springs at the top of the studs, it provides support and auxiliary restoring force, thereby ensuring that the TENG unit always performs stable reciprocating motion along the vertical axis. The base adopts a stepped design and is precisely assembled with the outer shell through edge grooves, enabling the sensor to be integrated as a sensing unit within the sealed cavity of the measurement-while-drilling instrument while ensuring overall structural stability. As the core unit of the sensor, a Z-shaped fiber paper structure is positioned between the sliding mass block and the base, with copper (Cu) and Kapton friction layers of 70 mm × 40 mm symmetrically attached to its surfaces. When external vibration excitation drives the mass block to produce vertical displacement, the Z-shaped structure undergoes periodic compression and release, enabling regulated contact-separation transitions between the Kapton and Cu surfaces. The Z-shaped folded structure increases the effective contact-separation area within a limited space, thereby enhancing the stability of the triboelectric output under low-frequency vibration. Meanwhile, the sliding mass block, guide studs, and spring structure constrain the motion direction, allowing the friction layers to achieve repeatable periodic contact and thus ensuring the stability and distinguishability of the output pulse signals from the TENG unit.

A permanent magnet, a spring, and a coil constitute the main body of the EMG. The power generation process is driven by external vibrations, which force the coil to undergo reciprocating motion relative to the stationary permanent magnet. This dynamic interaction effectively enables the periodic induction of electricity. This movement induces periodic variations in the magnetic flux penetrating the coil’s cross-section. In accordance with Faraday’s Law of Electromagnetic Induction, this variation in magnetic flux gives rise to an induced electromotive force across the coil. This mechanism effectively facilitates the transduction of mechanical kinetic energy into electrical output.

The TENG unit is responsible for vibration sensing, whereas power generation is achieved through the combined action of the TENG and EMG units. The operating principle of the TENG unit is described as follows. The sensor’s operational cycle, driven by the synergy of triboelectrification and electrostatic induction, manifests as four sequential stages (Steps 1–4) when subjected to fracturing vibration loads, as illustrated in Figure 1b. Initially, the vibrating body is triggered by the vibration of downhole drilling tools, at which point the Kapton film and Cu come into physical contact under external excitation (Step 1). Owing to the disparity in electron affinities, the Cu–Kapton interface experiences an instantaneous charge transfer during contact. As Cu possesses a higher propensity for electron loss, equivalent amounts of opposing charges accumulate on the Cu and Kapton surfaces. Subsequently, driven by the spring’s restorative force, the contact surfaces begin to disengage (Step 2). This movement induces an electron migration from the Cu layer to the back electrode via the outer loop, yielding a transient current pulse. Upon attaining the furthest clearance between tribo-layers (Step 3), charge distribution stabilizes, thereby halting electron migration and neutralizing the output current. As the stored potential energy of the springs is released, The gap between the triboelectric layers steadily narrows (Step 4), and the reduction in potential difference prompts the reverse transfer of charges, consequently yielding an opposite current spike within the loop. Over the course of a full fracturing-induced vibration sequence, the TENG continuously generates voltage pulses and alternating current signals. By quantifying the pulse frequency and peak signal intensity, precise monitoring of the vibration frequency is enabled.

In this electromagnetic energy harvesting system, the coil suspended within the elastic support structure undergoes vertical reciprocating motion relative to the permanent magnet under external excitation. The electromagnetic transduction process can be divided into three stages, as illustrated in Figure 1c. First, during the descending phase, as the coil approaches the permanent magnet, the magnetic flux penetrating the coil cross-section increases monotonically with the enhancement of the magnetic field intensity. According to Lenz’s Law, an induced current is generated to create an opposing magnetic field, manifesting as a current deflection in a specific direction. Subsequently, when the coil reaches the limit displacement of the spring extension, its instantaneous velocity drops to zero. At this point, although the magnetic flux peaks at this stage, the time rate of change in the flux is zero. Consequently, the induced electromotive force disappears, and the output current returns to the zero-level. Finally, driven by the linear restoring force of the spring, the coil bounces upward away from the magnet. During this departure, the magnetic flux continuously diminishes, leading to a polarity reversal in the rate of flux change. This induces a current component with a polarity opposite to that observed during the approach phase Through this repetitive cycle, the system achieves the continuous conversion of mechanical vibrational energy into alternating electrical power.

## 3. Experimental Assessment and Results Analysis

### 3.1. Experimental Apparatus and Procedures

**Figure 2:**
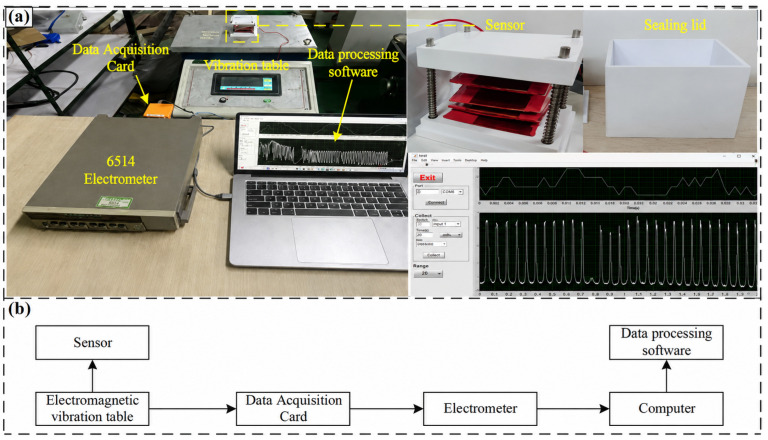
Experimental apparatus and workflow. (**a**) Sensor performance testing platform; (**b**) Experimental flow chart of the sensor.

The experimental setup used for sensor performance evaluation is shown in Figure 2a. Among these, the electromagnetic shaker is utilized to simulate the complex vibration frequencies and amplitudes of the downhole environment. The signal acquisition process, depicted in Figure 2b, involves the sequential processing of sensor outputs. The raw signals are first refined using a 6514 electrometer, then converted by a DAQ card, and finally transmitted to a computer for live graphical monitoring, and are finally integrated into the host computer system to achieve real-time signal monitoring, data analysis, and storage.

### 3.2. Output Characteristics Testing

**Figure 3:**
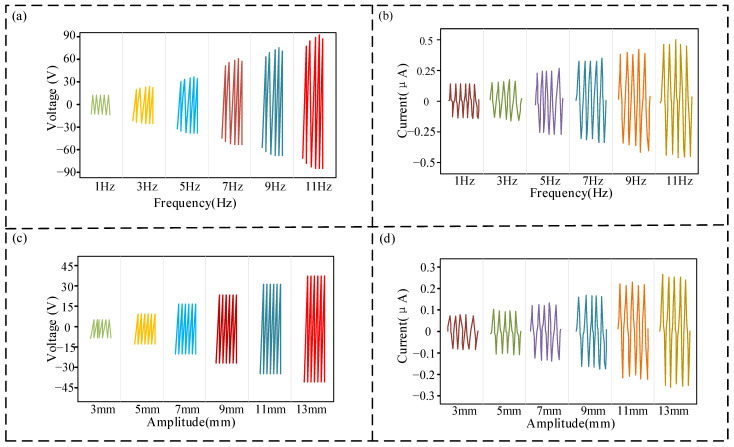
Sensor output characteristics. (**a**) Voltage waveforms at different frequencies; (**b**) current signals across a range of frequencies; (**c**) voltage responses to varying amplitudes; (**d**) Current signals at various amplitudes.

The performance characterization of the sensor primarily focuses on the output signal characteristics under varying frequencies and amplitudes, with the recorded data visualized in Figure 3. To optimize the experimental workflow while considering the data consistency across the multi-layered structures of the sensor, performance characterization was concentrated on the first layer as a proxy to ensure data reliability and streamline the measurement procedure. This approach accounts for the structural uniformity across all layers.

First, to investigate the impact of frequency on output characteristics, the amplitude of the electromagnetic shaker was fixed at 10 mm as a control variable. As the vibration frequency increases from 1 to 11 Hz, a pronounced upward trend is observed in both the output potential and current, as depicted in Figure 3a,b. The device achieves its peak performance at 11 Hz, yielding a maximum output voltage of 88.6 V and a current of 0.47 μA. Since the sensor measures the vibration frequency mainly by counting the number of voltage pulses per unit time, the frequency measurement sensitivity is defined as the ratio of the change in the measured frequency to the change in the externally applied vibration frequency, namely:

*Sf* = ∆*fp*/∆*fv* (1)

where *fp* is the output pulse frequency, namely the number of pulses per unit time; *fv* is the external vibration frequency; and *Sf* is the frequency response sensitivity. Within this range, the output pulse frequency of the sensor maintains an approximately one-to-one correspondence with the external vibration frequency. Therefore, its frequency measurement sensitivity is about 1.00, indicating that for every 1 Hz increase in the external vibration frequency, the pulse frequency identified by the sensor also increases by approximately 1 Hz. This demonstrates that the sensor has good low-frequency vibration sensing capability.

The results indicate that, within the range of 0–11 Hz, the vibration frequency is positively correlated with the number of output pulses. The core mechanism is that an increase in vibration frequency enhances the collision frequency between the friction layers per unit time, thereby accelerating the charge transfer rate and increasing the current. Concurrently, intensified charge migration is triggered by the stronger contact pressure across the interactive surfaces, a direct consequence of frequency-driven acceleration. This leads to enhanced frictional engagement, which contributes to the observed rise in voltage. However, once the frequency exceeds 11 Hz, the output signals gradually become disordered, and the synchronization between pulse counting and frequency disappears. Consequently, the detection bandwidth of the sensor encompasses frequencies from 0 to 11 Hz. This study mainly focuses on monitoring the low-frequency rotation-induced vibrations of the drilling assembly during coal-mine fracturing and drilling, rather than high-frequency impact-type vibration signals. Therefore, this frequency band can cover the main vibration range in the target application scenario and is thus suitable for practical use.

With the frequency maintained at a steady 3 Hz, the amplitude-dependent response of the sensor was subsequently investigated, as shown in Figure 3c,d. As the vibration displacement increases from 3 mm to 13 mm, the electrical output rises steadily, reaching a peak voltage of 37.6 V and a current of 0.25 μA at the maximum amplitude. The underlying principle resides in the control mechanism of the testing platform: amplifying the displacement elevates the acceleration, which augments the contact force between the material layers, leading to a more effective frictional contact, thereby increasing the output signal amplitude. Nevertheless, since the sensor measures vibration frequency by counting signal pulses, the variation in signal amplitude does not alter the pulse count, and therefore has no impact on the measurement accuracy of the frequency. Furthermore, because the voltage signal is at the volt (V) level—significantly higher than the microampere (μA) level of the current signal—it exhibits superior anti-interference capability. Thus, the voltage pulse signal served as the benchmark for our output measurements.

### 3.3. Measurement Error Testing

**Figure 4:**
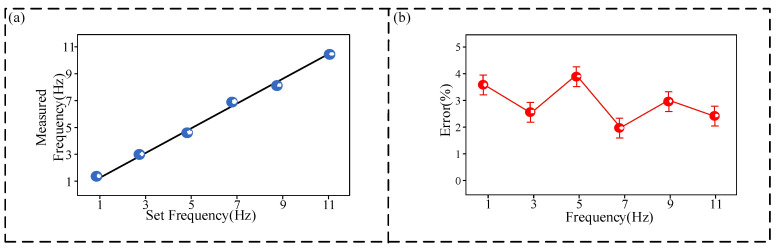
Experimental results of sensor measurement error. (**a**) Frequency calibration curve; (**b**) Measurement error plot.

To validate the measurement accuracy of the sensor, calibration tests were conducted on its frequency response characteristics, with the results presented in Figure 4. The output voltage signal of the sensor was acquired using a microcontroller-based data acquisition system at a sampling rate of 50 Sa/s. A dual-threshold level discrimination method was used for effective pulse identification: when the output voltage increased to 2.0 V or above, one valid pulse was recorded; when the output voltage decreased below 0.8 V, the counting state was reset; within the range of 0.8–2.0 V, the system maintained its previous logic state to reduce repeated counting caused by voltage fluctuations near the threshold. The output frequency was calculated from the time intervals of the valid pulse sequence and compared with the preset input frequency to evaluate the frequency recognition accuracy of the sensor. Since this study mainly focuses on the number of pulses and pulse intervals under low-frequency vibration, rather than the precise reconstruction of the transient waveform of individual pulses, no additional analog or digital filtering was applied to the raw output signal. The calibration curve in Figure 4a demonstrates a remarkably high linear correlation between the set frequency and the measured frequency, confirming a stable and quantifiable mapping relationship between the two parameters. Further analysis of the error distribution curve in Figure 4b reveals that across the testing range of 0–11 Hz, the sensor’s measurement error exhibits excellent stability, with no significant fluctuations observed as the frequency varies. Notably, the maximum measurement error consistently remains within 5%.

### 3.4. Evaluation of Power Generation Performance

**Figure 5:**
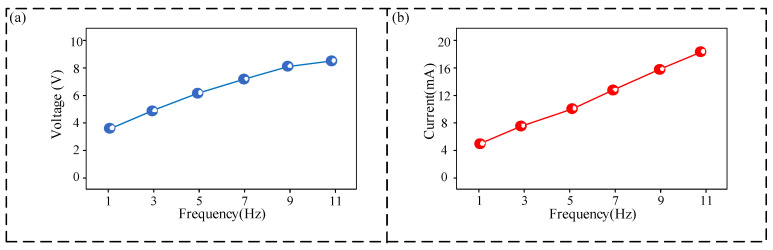
Output characteristics of the EMG part. (**a**) Fitted curves of output voltage at different vibration frequencies. (**b**) Fitted curves of output current at different vibration frequencies.

The evaluation of the energy-harvesting performance of the electromagnetic generator (EMG) module focused on its output characteristics at different vibration frequencies. As illustrated in Figure 5, both the output voltage and current exhibit a distinct linear upward trend as the frequency increases. Specifically, the output voltage was measured in the range of 3.8 V to 8.5 V, while the output current varied between 4.6 mA and 18.3 mA. The underlying mechanism for this phenomenon is that higher vibration frequencies directly enhance the reciprocating velocity of the coil within the magnetic field, thereby augmenting the rate of magnetic flux change. Consistent with Faraday’s law of induction, the induced electromotive force increases with the rate of flux change, which explains the superior power generation capability observed at higher frequencies.

Because the sensor operates through the coupled effects of contact electrification and electrostatic induction, the sensing process is inherently accompanied by energy generation. By capturing the continuous mechanical vibrations of downhole drilling tools, the internal friction layers are driven into high-frequency contact-separation motions. This movement stimulates cyclical carrier migration between the conductive layers, producing an alternating output in the external circuit and facilitating the conversion of mechanical work.

**Figure 6:**
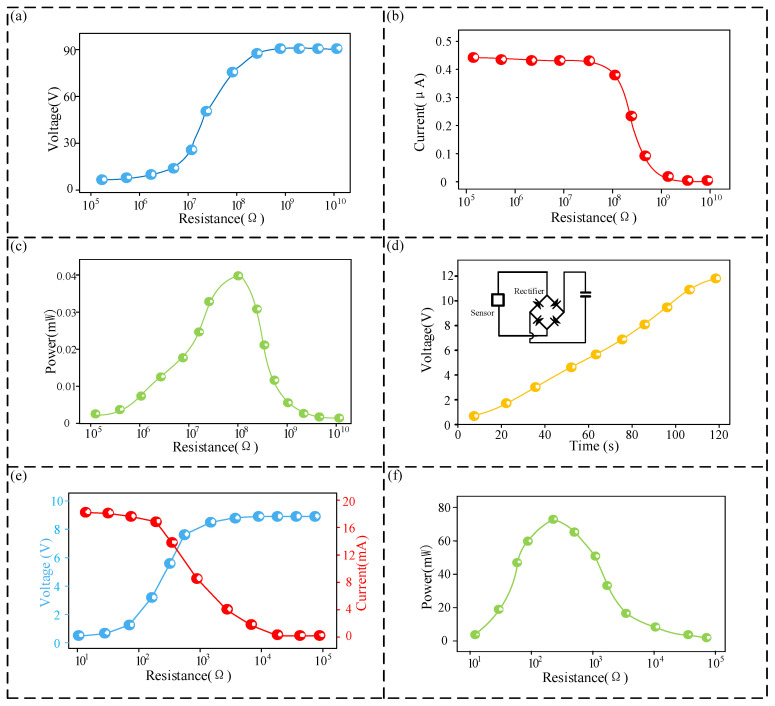
Power generation performance of the sensor. (**a**) TENG output voltage under varying load resistances; (**b**) TENG output current relative to load resistance; (**c**) Dependence of TENG output power on load resistance; (**d**) Charging behavior of the sensor’s TENG component; (**e**) EMG output voltage and current versus load resistance; (**f**) EMG output power as a function of load resistance.

The sensor consists of a triboelectric nanogenerator module (TENG) and an electromagnetic generator module (EMG), which convert mechanical energy into electrical energy under vibration excitation through the triboelectrification–electrostatic induction effect and the electromagnetic induction effect, respectively. To distinguish the output characteristics of the two energy-harvesting units, the electrical outputs of the TENG and EMG were tested separately under different external load resistances. Figure 6a–d present the output characteristics of the TENG, while Figure 6e,f show those of the EMG. Except for the capacitor charging experiment shown in Figure 6d, the load tests were conducted by directly measuring the AC output signals under the corresponding external resistance conditions. The power values were calculated from the voltage and current measured under each load condition and are expressed as peak output power.

For the TENG, the electrical signal originates from the periodic contact and separation between the triboelectric layers under vibration excitation. This process induces periodic charge generation and transfer on the surfaces of the triboelectric layers, thereby producing an AC output in the external circuit through electrostatic induction. To evaluate the load-dependent output characteristics of the TENG, its output voltage and current were measured over an external resistance range from short-circuit conditions to 10^10^ Ω. As shown in Figure 6a,b, with increasing load resistance, the output voltage of the TENG increases nonlinearly and gradually approaches saturation, with a maximum output voltage of approximately 90 V. In contrast, the output current decreases with increasing load resistance, and the maximum short-circuit current is approximately 0.45 μA. The output power was calculated from the voltage and current under different load conditions, as shown in Figure 6c. When the external load resistance was 10^8^ Ω, the TENG reached a favorable load-matching condition, with a peak output power of approximately 0.04 mW. Furthermore, in the capacitor charging test, the rectified TENG output was connected to a 1 μF capacitor. As shown in Figure 6d, the module charged the capacitor to approximately 12 V within 120 s, indicating that the TENG has a certain capability for energy harvesting and storage.

For the EMG, the output signal is mainly generated by the variation in magnetic flux caused by the relative motion between the coil and the magnet, producing an induced electromotive force based on electromagnetic induction. As shown in Figure 6e,f, the output characteristics of the EMG are also affected by the external load resistance. As the load resistance increases, the output voltage of the EMG gradually increases, while the output current correspondingly decreases. The power calculated from the output voltage and current under different load conditions indicates that, when the external load resistance is 3.3 × 10^2^ Ω, favorable impedance matching is achieved between the EMG and the load, and the peak output power reaches 110.5 mW.

### 3.5. Environmental Adaptability Testing

**Figure 7:**
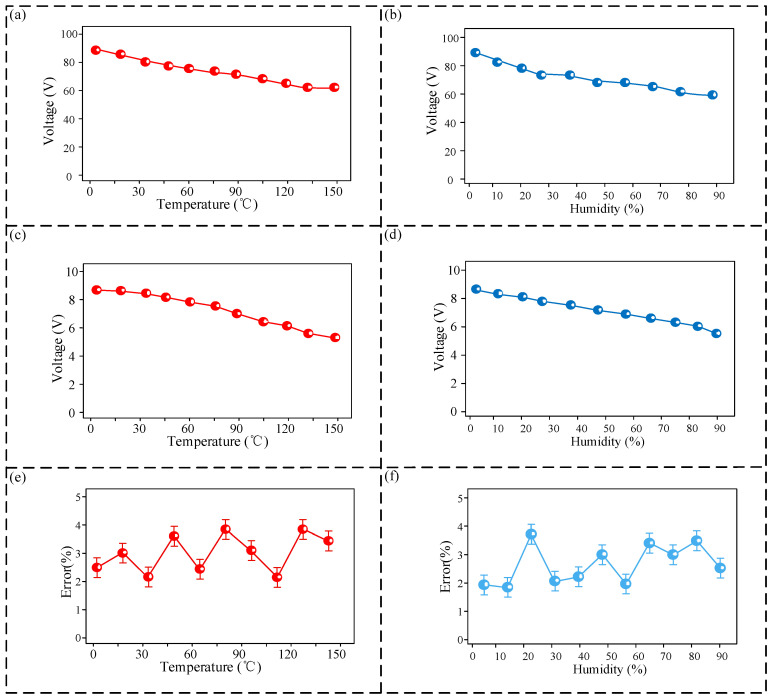
Performance evaluation of the sensor under varied environmental conditions. (**a**) Temperature-dependent TENG output voltage; (**b**) TENG output response to varying relative humidity (RH); (**c**) EMG output voltage at different temperatures; (**d**) Influence of relative humidity (RH) on EMG output; (**e**) Frequency recognition errors of the TENG at different temperatures; (**f**) Frequency recognition errors of the TENG at different humidity levels.

Downhole environments are characterized by significant fluctuations in temperature and humidity, which impose strict requirements on the performance stability of vibration sensors. To assess the operational robustness of the developed sensor under diverse conditions, a series of influence-factor characterizations were performed. As shown in Figure 7a, within the temperature range below 150 °C, A subtle negative correlation is observed between the output voltage and ambient temperature, characterized by a marginal decline as the temperature rises, with a total reduction of approximately 28%. This indicates that although elevated temperatures somewhat inhibit charge transfer, the sensor demonstrates good thermal stability within the target temperature range. The dependence of the sensor’s performance on relative humidity (RH) is depicted in Figure 7b, revealing a progressive decline in output voltage as the RH level rises from 30% to 90%. Such a phenomenon largely stems from water molecules acting as a shield for the friction layer’s surface charges in humid environments, thereby inhibiting the charge transfer process. Nevertheless, the sensor maintains effective signal output even under extremely high humidity conditions. As shown in Figure 7c,d, the output voltage variations in the TENG under different temperature and humidity conditions are presented, respectively. The results indicate that even under extreme test conditions of 150 °C and 90% RH, the output voltage of the sensor remains significantly higher than the 2 V TTL recognition threshold, demonstrating that it can still generate pulse signals that can be effectively recognized by the microprocessor. Furthermore, as shown in Figure 7e,f, the frequency recognition errors of the TENG under different temperature and humidity conditions are all below 5%. These results indicate that although variations in temperature and humidity have a certain influence on the output voltage, the sensor can still maintain high signal recognition accuracy and good environmental adaptability.

**Figure 8:**
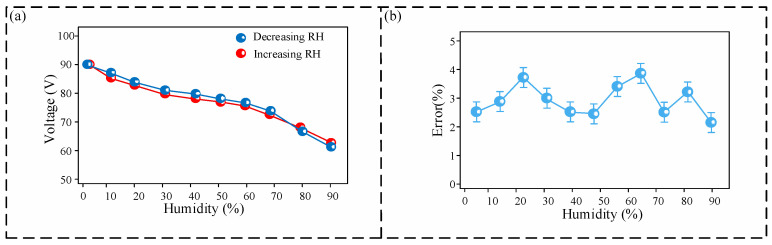
Humidity hysteresis characteristics of the TENG unit at different RH levels: (**a**) Output voltage during humidification and dehumidification; (**b**) Corresponding hysteresis error.

To evaluate the effect of changes in ambient humidity on the output performance of the TENG unit, the output voltage was measured separately during the processes of increasing and decreasing relative humidity. As shown in Figure 8a, the output voltage of the TENG unit exhibits an overall gradual decline with increasing relative humidity. This is mainly because, under high-humidity conditions, water molecules are more readily adsorbed onto the surface of the triboelectric materials, which aggravates interfacial charge dissipation, weakens the surface charge retention capability, and ultimately leads to a reduced output signal. Meanwhile, the output curves corresponding to the humidification and dehumidification processes remain generally close to each other over the entire tested humidity range, with only slight deviations observed at a few humidity points, indicating that the device exhibits only a weak hysteresis effect during humidity variation. To further quantitatively characterize the humidity hysteresis, the output differences between the humidification and dehumidification processes at the same relative humidity were analyzed, and the corresponding humidity hysteresis error was obtained, as shown in Figure 8b. The results show that the humidity hysteresis error remains within 5% throughout the entire test range, demonstrating that the TENG unit has weak humidity hysteresis characteristics and good reversibility of output response during the humidification and dehumidification processes.

**Figure 9:**
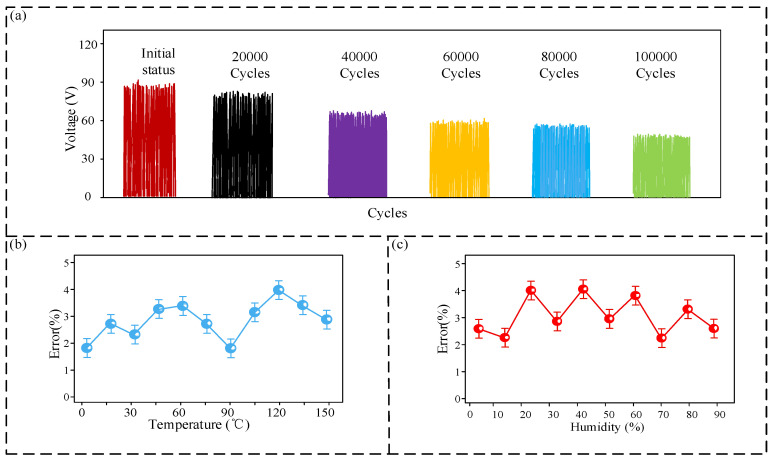
Long-term stability test of the sensor. (**a**) Output voltage under different operation cycles. (**b**) Frequency recognition errors at different temperatures after 100,000 cycles. (**c**) Frequency recognition errors at different humidity levels after 100,000 cycles.

Additionally, Figure 9 presents the long-term stability test results of the sensor. As shown in Figure 9a, after 100,000 consecutive vibration cycles, the sensor still retained approximately 64% of its initial output, with an output voltage of 57.6 V, which remained significantly higher than the 2 V TTL recognition threshold. This indicates that the proposed Z-shaped hybrid TENG–EMG sensor possesses good cycling stability and mechanical durability. Furthermore, as shown in Figure 9b,c, after 100,000 vibration cycles, the frequency recognition errors of the sensor under different temperature and humidity conditions remained below 5%. These results demonstrate that the sensor can maintain high signal recognition accuracy and good environmental adaptability even after long-term operation.

The sensor measures vibration frequency by relying on voltage pulse signals, which are directly read by a microprocessor via its pulse input port. Pulse input interfaces of microprocessors generally adhere to the Transistor–Transistor Logic (TTL) specifications. Under this convention, a signal is recognized as a valid input provided its voltage magnitude surpasses a threshold of approximately 2 V, the microprocessor can accurately identify the signal. The experimental results in Figure 9 indicate that despite the reduction in output voltage due to temperature, humidity, and long-term cycling, the voltage amplitudes remain significantly higher than the 2 V threshold. This ensures compliance with TTL recognition standards. Accordingly, the functional window of the sensor is constrained to temperatures under 150 °C and a relative humidity level not exceeding 90%.

### 3.6. Comparison and Discussion of Existing Vibration Sensing Technologies

To further illustrate the performance characteristics of the proposed sensor, it was compared with several typical vibration sensors. As shown in Table 1, conventional electromagnetic geophones, MEMS accelerometers, piezoelectric accelerometers, and fiber-optic vibration sensors exhibit certain advantages in terms of measurable frequency range and measurement accuracy, with measurable frequency ranges of 1–600 Hz, 1 kHz, 0.4–14 kHz, and 0–60 Hz, respectively, and corresponding errors of approximately 5%, 0.1%, 1%, and 2.03%. However, none of these sensors are self-powered and they still mainly rely on external batteries or cable-based power supply, making it difficult to simultaneously meet the demands of long-term online underground monitoring and energy harvesting.

The sensor proposed in this work is mainly intended for low-frequency vibration monitoring under complex underground coal mine conditions. It can achieve effective measurement in the range of 0–11 Hz with a measurement error of less than 5%, which is sufficient for underground low-frequency vibration monitoring. Compared with existing self-powered TENG vibration sensors, the proposed sensor shows essentially the same measurement range and error level, as both can realize low-frequency vibration measurement in the range of 0–11 Hz with an error below 5%. However, in terms of output performance, the typical output power of the proposed sensor reaches 110.5 mW, which is significantly higher than the 32.4 × 10^−9^ W of existing self-powered TENG vibration sensors, demonstrating a much stronger energy-harvesting capability. Therefore, while maintaining low-frequency vibration measurement capability and good measurement accuracy, the proposed sensor also integrates self-powering and energy harvesting functions, showing greater application potential for long-term online monitoring in the complex underground environment of coal mines.

## 4. Conclusions

In this study, a Z-shaped triboelectric vibration sensor was developed as a self-powered monitoring system for coal mine fracturing applications. The sensor exhibits robust performance in high-temperature (up to 150 °C) and high-moisture (up to 90% RH) environments. Performance-wise, it exhibits a maximum output capacity reaching 90 V and 0.5 μA under optimal conditions, and keeps measurement errors within 5%. Under a matched load of 10^8^ Ω, its maximum instantaneous output power reaches 0.04 mW. The performance of the EMG was systematically evaluated across various external loads. It is found that impedance matching is attained when the load resistance is set to 3.3 × 10^2^ Ω, which corresponds to a maximum output power of 110.5 mW.

The primary novelty of this sensor is its autonomous power generation, which obviates the necessity for any auxiliary energy sources. This effectively reduces the dependence of downhole monitoring equipment on traditional batteries, thereby significantly mitigating the loss of drilling efficiency and profitability associated with frequent battery replacements. In addition, the electricity harvested by the device can be buffered in real-time. Upon reaching a requisite threshold, the stored charge can be discharged to energize auxiliary downhole electronics, such as measurement-while-drilling tools.

However, the electrical output generated by the sensor fluctuates within the milliwatt range, which is insufficient for directly powering high-consumption devices. Future research will focus on three areas: (1) optimizing structural designs to scavenge multiple mechanical energy sources, including downhole vibrations, mud flow, and drill-bit rotation, thereby increasing the total power output; (2) enhancing the energy conversion efficiency per unit area by using high-performance triboelectric and electromagnetic materials; and (3) improving low-frequency measurement accuracy through optimization of the spring stiffness, slider mass, structural alignment, and signal-processing algorithms.

## Figures and Tables

**Table 1:** Performance comparison of different underground vibration monitoring sensors.

Type	Measurable Frequency Range	Typical Error/Accuracy	Self-Powered Capability	Typical Output Power
Electromagnetic geophone	1–600 Hz	5%	No	Not applicable
MEMS accelerometer	1 kHz	0.1%	No	Not applicable
Piezoelectric accelerometer	0.4–14 kHz	1%	No	Not applicable
Fiber-optic vibration sensor (FBG)	0–60 Hz	2.03%	No	Not applicable
Self-powered TENG vibration sensor	0–11 Hz	<5%	Yes	32.4 × 10^−9^ W
Sensor proposed in this work	0–11 Hz	<5%	Yes	110.5 mW

## Data Availability

The original contributions presented in the study are included in the article, further inquiries can be directed to the corresponding author.
